# COMPARISON OF QUALITY INDICATORS IN RURAL AND URBAN ASSISTED LIVING FACILITIES

**DOI:** 10.1093/geroni/igae098.2995

**Published:** 2024-12-31

**Authors:** Alexa Stuifbergen, Stephanie Morgan, Katie Trainum, Heather Becker

**Affiliations:** The University of Texas Austin, Austin, Texas, United States; The University of Texas Austin, Austin, Texas, United States; The University of Texas Austin, Austin, Texas, United States; The University of Texas Austin, Austin, Texas, United States

## Abstract

As the population of older adults grows, the demand for assisted living facilities (ALFs) is expected to increase. This growth will disproportionately affect rural ALFs, as the proportion of older adults living in rural areas is growing at a faster rate than urban areas, and older adults living in rural areas are in poorer health compared to their urban counterparts. This exploratory descriptive study examined differences between facility characteristics and resident perspectives of care between rural and urban ALFs using data collected as part of a major statewide assessment of quality of care in ALFs. The research team conducted site visits to collect facility level data from staff and resident interviews regarding perspectives of quality of care. The sample included 517 facilities from 8 geographic regions of a large Southwestern state with 143 of the facilities located in rural settings. Overall, licensed bed size ranged from 3 to 177 with the number of occupied beds significantly greater in urban ALFs (Mean=33.71, SD 28.51) than in rural ALFS (Mean=22.54, SD 15.93) (t=5.37, p<.001). Overall, residents rated their health as good, very good, or excellent (78%) although a greater percentage of rural residents (23%) rated their health as fair or poor compared to urban residents (16%). Resident satisfaction with quality of health care and quality of experience in the facility was higher among residents of rural vs. urban ALFs. Additional research is needed to more fully explore factors influencing quality of life and quality of care in rural and urban ALFs.

